# Dataset on graphite nanoplatelet enhanced HDPE composites: An ensemble machine learning approach estimating the tensile modulus, toughness, and hardness thus creating a roadmap for new product development

**DOI:** 10.1016/j.dib.2024.110987

**Published:** 2024-10-09

**Authors:** Nitesh Dhar Badgayan, Santosh Kumar Sahu, Avisek Kundu, Seeboli Ghosh Kundu

**Affiliations:** a Symbiosis Centre for Management Studies, Bengaluru Campus, Symbiosis International (Deemed University), Pune, India; b School of Mechanical Engineering, VIT-AP University, Besides A.P. Secretariat, Amaravati 522237, Andhra Pradesh, India; c Technology Consulting (Data Science, ML & AI), Ernst & Young LLP and Research Scholar, Department of Operations and IT, IBS, Hyderabad (A Constituent of ICFAI Foundation for Higher Education), Hyderabad, India

**Keywords:** Mechanical properties, Machine learning, Random forest, Polymer composite, Graphite

## Abstract

This article presents a dataset examining the impact of Graphite Nanoplatelets (GNP), injection pressure, and injection temperature on the mechanical properties of High-Density Polyethylene (HDPE) composites. The study uses an ensemble machine learning approach to build a predictive model for response variables: tensile modulus, toughness, and hardness. The model used was a Random Forest regressor, which was robust to outliers and data distribution types. Grid Search CV and Random Search CV were used for hyperparameter tuning, and predictive analysis of model parameters was conducted using both. The response variables were divided into high, medium, and low classes based on the quartile distributions. A Decision Tree classifier was used to derive rules for each class. These methods and the comprehensive dataset and analysis offer a robust foundation for future research in polymer composites. The insights can be leveraged with further research to develop the highest grade of polymer composite products. This might include new applications, improved performance, or cost reduction by optimizing the resources. This research may help in developing a detailed concept for the viable ideas and testing of a new polymer-based product thus bringing a significant impact in the polymer industry as a whole.

Specifications Table

**Table:** 

Subject	Data Science
Specific subject area	Machine Learning Driven Data Analytics
Type of data	Raw Data, Analysed Data, SEM Images, Figures, Graphs and Codes.
Data collection	HDPE composite was prepared using an injection molding technique with the variegated percentage of GNP and varying the injection pressure and temperature. The samples for tensile testing were prepared according to ASTM D1708 standard, and tensile properties were evaluated using a Universal Tensile Testing Machine. Similarly Vickers hardness tester was used to assess hardness*.*
Data source location	Experiments were conducted at the School of Mechanical Engineering, VIT AP University, CIPET Bhubaneswar, and NIST University, Berhampur.
Data accessibility	Repository name: Mendeley Data Data identification number: (10.17632/4h98rz9f92.3) Direct URL to data: https://data.mendeley.com/datasets/4h98rz9f92/3

## 1 Value of the Data

• The dataset of the influence of graphene, pressure, and temperature on tensile modulus, toughness, and hardness of High-Density Polyethylene provides a comprehensive view of data of injection molding of HDPE, thereby enabling robust statistical and machine learning analysis.
• The two datasets, as shown in Table 1, Table 2, expound details of input parameters (graphite nanoplatelet %, injection pressure in MPa, and injection temperature in degrees Celsius). Table 2 categorizes the output parameters tensile modulus, toughness and hardness into three classes/categories: High, Medium, and Low taking quartiles.

**Table 1:** Input parameters.

Expt. No.	GNP %	Temperature (°C)	Pressure (MPa)
1	0	170	22.2
2	0	170	29.1
3	0	170	31.4
4	0	170	33.1
5	0	170	34.8
6	0	170	22.5
7	0	190	27
8	0	190	31.3
9	0	190	33.5
10	0	190	37
11	0.01	190	20
12	0.01	190	28
13	0.01	190	32.2
14	0.01	190	37.5
15	0.01	190	27.2
16	0.02	190	29.9
17	0.02	190	35
18	0.02	190	38.4
19	0.02	210	36.1
20	0.02	210	35.8
21	0.03	210	22.1
22	0.03	210	29.1
23	0.03	210	31.4
24	0.03	210	33.7
25	0.03	210	34.4
26	0.04	210	22.6
27	0.04	180	29.5
28	0.04	180	31.7
29	0.04	180	33.2
30	0.04	180	34.9
31	0.05	180	22.3
32	0.05	180	28.1
33	0.05	180	31.3
34	0.05	180	33.7
35	0.05	180	35.6

**Table 2:** Output parameters.

Expt. No.	Tensile modulus (GPa)	Toughness (MPa)	Hardness (HV)
1	1.01	302.41	116.43
2	1.02	301.43	115.45
3	1.02	302.12	116.35
4	1.01	302.34	114.21
5	1.01	305.11	113.34
6	1.02	301.74	114.68
7	1.01	302.11	115.72
8	1.03	304.87	114.41
9	1.02	302.91	115.81
10	1.01	303.21	116.01
11	1.05	323.41	120.43
12	1.06	325.43	121.45
13	1.06	325.12	120.35
14	1.05	328.34	124.21
15	1.06	329.11	126.34
16	1.05	328.72	125.21
17	1.06	329.66	126.14
18	1.06	330.71	124.43
19	1.06	329.12	125.41
20	1.06	328.69	126.34
21	1.09	340.82	125.67
22	1.11	341.78	124.42
23	1.10	342.75	125.43
24	1.11	341.53	124.78
25	1.09	342.88	134.67
26	1.12	353.28	137.68
27	1.12	354.53	137.81
28	1.13	355.45	138.81
29	1.12	355.21	138.21
30	1.12	355.12	137.71
31	1.12	358.12	139.91
32	1.13	359.21	140.43
33	1.14	358.32	140.21
34	1.13	358.43	140.44
35	1.13	359.65	140.91
• The dataset can be helpful for future researchers in the following aspects.
• The data serves as a reference for comparative studies. It may help researchers develop new composite materials and allow them to understand how processing parameters affect material properties.
• It is a reference for algorithm benchmarking to help researchers determine the most effective and efficient method for predicting material properties.

## 2 Background

The objective of the article is to present a comprehensive experimental dataset that captures the mechanical properties of graphene-reinforced HDPE with varying injection pressure and injection temperature; the dataset was generated through melt mixing and intercalation method using injection molding, and it serves the following objectives

• To determine the effect of GNP, pressure, and temperature on mechanical properties of HDPE nanocomposite.
• To identify which is the most significant parameter that has the maximum influence on mechanical properties
• Predictive modeling of mechanical properties using ensemble machine learning algorithm.
• Using a Decision Tree Classifier to establish rules for obtaining optimum property parameters

## 3 Data Description

The dataset used for analysis delineates three input parameters: GNP %, Injection Pressure, and Injection Temperature, and their effect on tensile modulus, toughness, and hardness. HDPE was the base matrix, and the GNP %, injection pressure and injection temperature were varied, as shown in Table 1. Table 2 details the measured tensile modulus, toughness, and hardness parameters. Table 3 presents the output parameters in categorical measures as High, Medium, and Low. Three quartile observations were used to categorize the response data to derive rules.

**Table 3:** Specification of materials.

Materials	Specification
HDPE	Pellet dimension 5 to 8 mm; MFI 0.7 g/10 min, Density 0.94 g/cm^3^.
GNP	Thickness 6-10 nm, Purity >99.9 %, Surface area (SA): 60-200 m^2^/g; Average lateral dimension=10μm; Density: 0.1 g/cm^3^.

## 4 Experimental Design, Materials and Methods

### 4.1 Materials

**Fig. 1:**
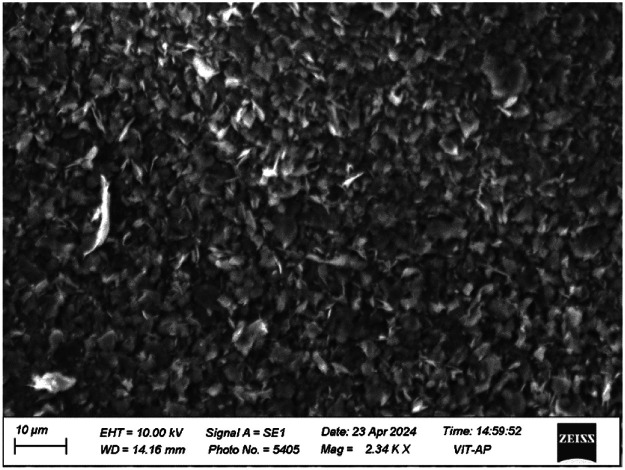
SEM image of GNP.

High density polyethylene (HDPE) pellets were purchased from Indian Oil Corporation Limited, India. Graphite nano-platelets (GNP) filler was purchased from Nano Research Elements, India. The detailed technical specifications for HDPE and GNP was shown in Table 3. The Scanning Electron Microscope (SEM) results, shown in Fig. 1, confirm that GNP has a platelet structure

### 4.2 Methods

#### 4.2.1 Fabrication of composites

**Fig. 2:**
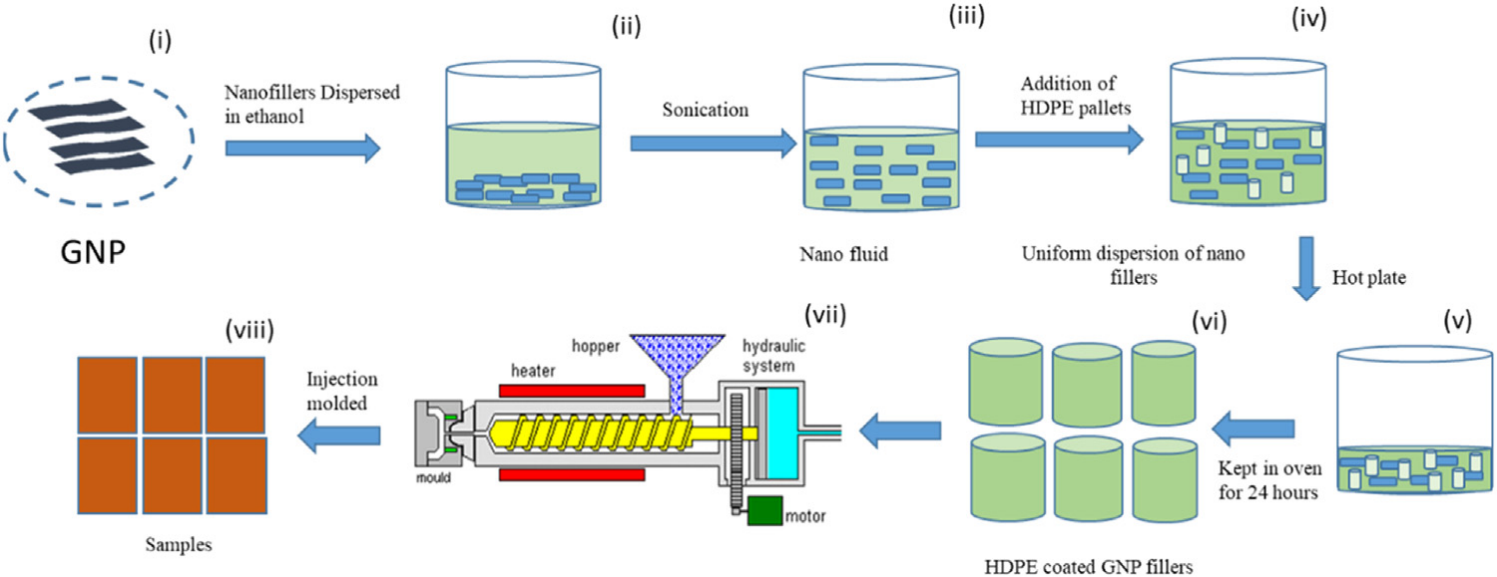
Fabrication route followed.

The fabrication process is depicted in Fig. 2 from steps (i) to (viii). First, the GNP nanoparticles undergo chemical modification [1,2]. The chemical modification comprises of required wt. % of Graphite Nano-platelets that were firstly immersed in ethanol and tetrahydrofuran for 24 h at room temperature, then it was washed with distilled water and finally dried at 100 ͦC in a vacuum oven for 24 h to get the functionalized GNP powder. After that, chemically modified GNP nanoparticles are placed in a beaker and combined with ethanol at a 1:0.5 ratio. This mixture is then magnetically stirred and sonicated to ensure even dispersion. The resulting nanofluid is blended with HDPE pellets and heated on a hot plate until all the ethanol has evaporated. The mixture is baked in an oven for 24 hours to eliminate any remaining moisture. The HDPE, now coated with GNP nanofillers, is introduced into an injection molding machine to create the HDPE/0.01 nanocomposite. By adjusting the injection molding parameters, such as temperature and pressure, various HDPE/GNP composite samples are fabricated, as detailed in Table 1 for further analysis, which is discussed in the next section.

### 4.3 Mechanical testing

**Fig. 3:**
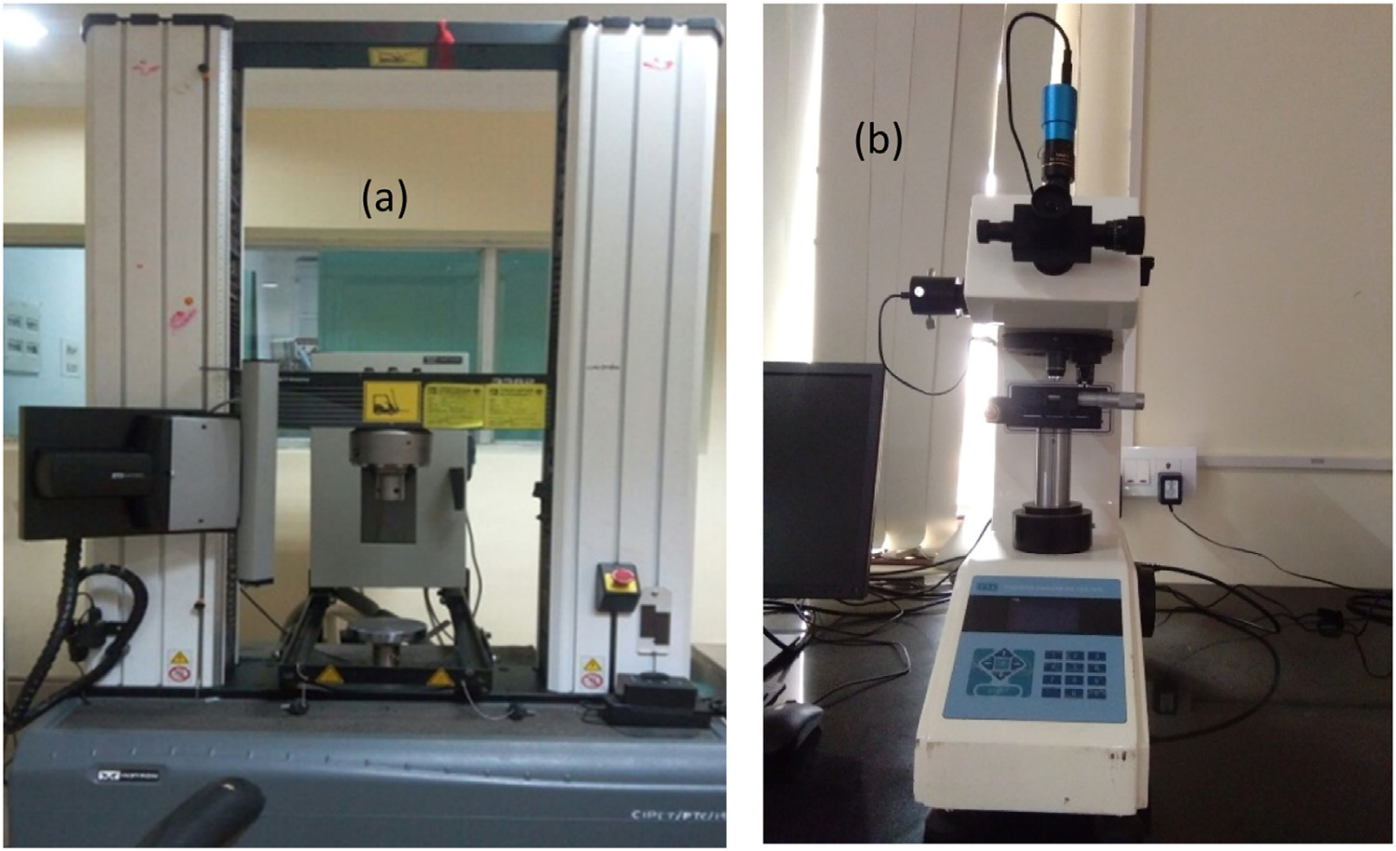
a) UTM; b) Hardness equipment used.

A tensile test was conducted according to ASTM D1708 standards to determine the tensile modulus and toughness by cutting the fabricated samples as described above [3,4]. An Instron 8801 Universal Testing Machine (UTM) with a dynamic load capacity of ±100 kN and a load cell accuracy of 0.005% was utilized. The crosshead speed was set and maintained at 1 mm/min throughout the test. For hardness evaluation, the FMV1-MC-AT Vickers indenter, equipped with a diamond pyramid indenter, was used. A load of 0.1 kg and a dwell time of 10 seconds was used. Each test was repeated five times to ensure accuracy. Fig. 3 shows the equipment used for the mechanical testing.

### 4.4 Machine learning methods

#### 4.4.1 OLS method

**Fig. 4:**
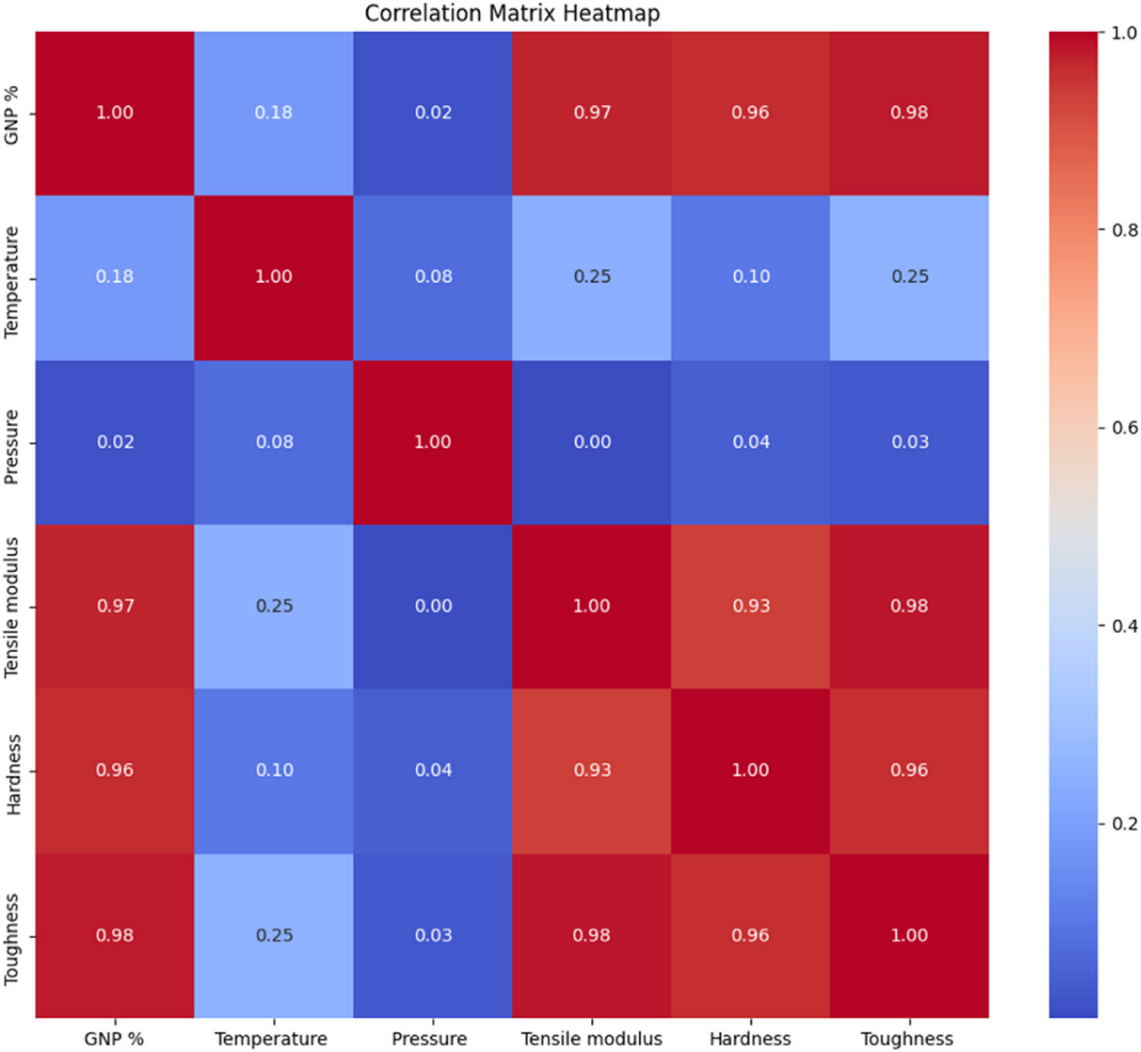
Correlation heatmap.

The Influence of GNP %, pressure, and temperature on tensile modulus, toughness and hardness were investigated using statistical tests [5,6], and a correlation heat map was done using Ordinary Least Square (OLS) method as shown in Fig. 4. It is also used to do prediction and is used to R^2^ and Adj. R^2^ and the interaction effect of each variable, as shown in Tables 4, 5 and 6.

**Table 4:** OLS regression results - tensile modulus.

R-squared:	0.957						
Adj. R-squared:	0.945						
F-statistic:	85.04						
Prob (F-statistic):	9.89e-17						
Log-Likelihood:	114.47						
AIC:	-212.9						
BIC:	-200.5						
		coef	Std err	t	P>|t|	[0.025	0.975]
Intercept		0.6463	0.300	2.155	0.040	0.031	1.262
Pressure		0.0106	0.010	1.072	0.293	-0.010	0.031
Temperature		0.0022	0.002	1.307	0.202	-0.001	0.006
Pressure: Temperature		-6.201e-05	5.44e-05	-1.140	0.264	-0.000	4.96e-05
Q(“GNP %”)		7.8110	10.861	0.719	0.478	-14.475	30.097
Pressure:Q(“GNP %”)		-0.1816	0.369	-0.491	0.627	-0.940	0.577
Temperature:Q(“GNP %”)		-0.0362	0.060	-0.609	0.548	-0.158	0.086
Pressure:Temperature:Q(“GNP %”)		0.0012	0.002	0.591	0.559	-0.003	0.005

**Table 5:** OLS regression results – toughness.

R-squared:	0.966						
Adj. R-squared:	0.957						
F-statistic:	109.7						
Prob (F-statistic):	3.74e-18						
Log-Likelihood:	-96.965						
AIC:	209.9						
BIC:	222.4						
		Coef	Std err	t	P>|t|	[0.025	0.975]
Intercept		109.7449	126.070	0.871	0.392	-148.929	368.419
Pressure		4.9068	4.150	1.182	0.247	-3.608	13.421
Temperature		1.0995	0.698	1.576	0.127	-0.332	2.531
Pressure: Temperature		-0.0275	0.023	-1.203	0.240	-0.074	0.019
Q(“GNP %”)		4819.8348	4564.950	1.056	0.300	-4546.67	1.42e+04
Pressure:Q(“GNP %”)		-88.4936	155.295	-0.570	0.573	-407.132	230.145
Temperature:Q(“GNP %”)		-21.8601	25.017	-0.874	0.390	-73.191	29.471
Pressure:Temperature:Q(“GNP %”)		0.5335	0.851	0.627	0.536	-1.212	2.279

**Table 6:** OLS regression results – hardness.

R-squared:	0.942						
Adj. R-squared:	0.927						
F-statistic:	62.73						
Prob (F-statistic):	4.73e-15						
Log-Likelihood:	-78.253						
AIC:	172.5						
BIC:	184.9						
		Coef	Std err	T	P>|t|	[0.025	0.975]
Intercept		197.9218	73.862	2.680	0.012	46.369	349.475
Pressure		-3.0474	2.431	-1.253	0.221	-8.036	1.941
Temperature		-0.4540	0.409	-1.111	0.276	-1.292	0.385
Pressure: Temperature		0.0168	0.013	1.257	0.220	-0.011	0.044
Q(“GNP %”)		-2309.5058	2674.538	-0.864	0.395	-7797.205	3178.194
Pressure:Q(“GNP %”)		123.5749	90.985	1.358	0.186	-63.111	310.261
Temperature:Q(“GNP %”)		15.2955	14.657	1.044	0.306	-14.779	45.370
Pressure:Temperature:Q(“GNP %”)		-0.6730	0.498	-1.350	0.188	-1.696	0.350

**Fig. 5:**
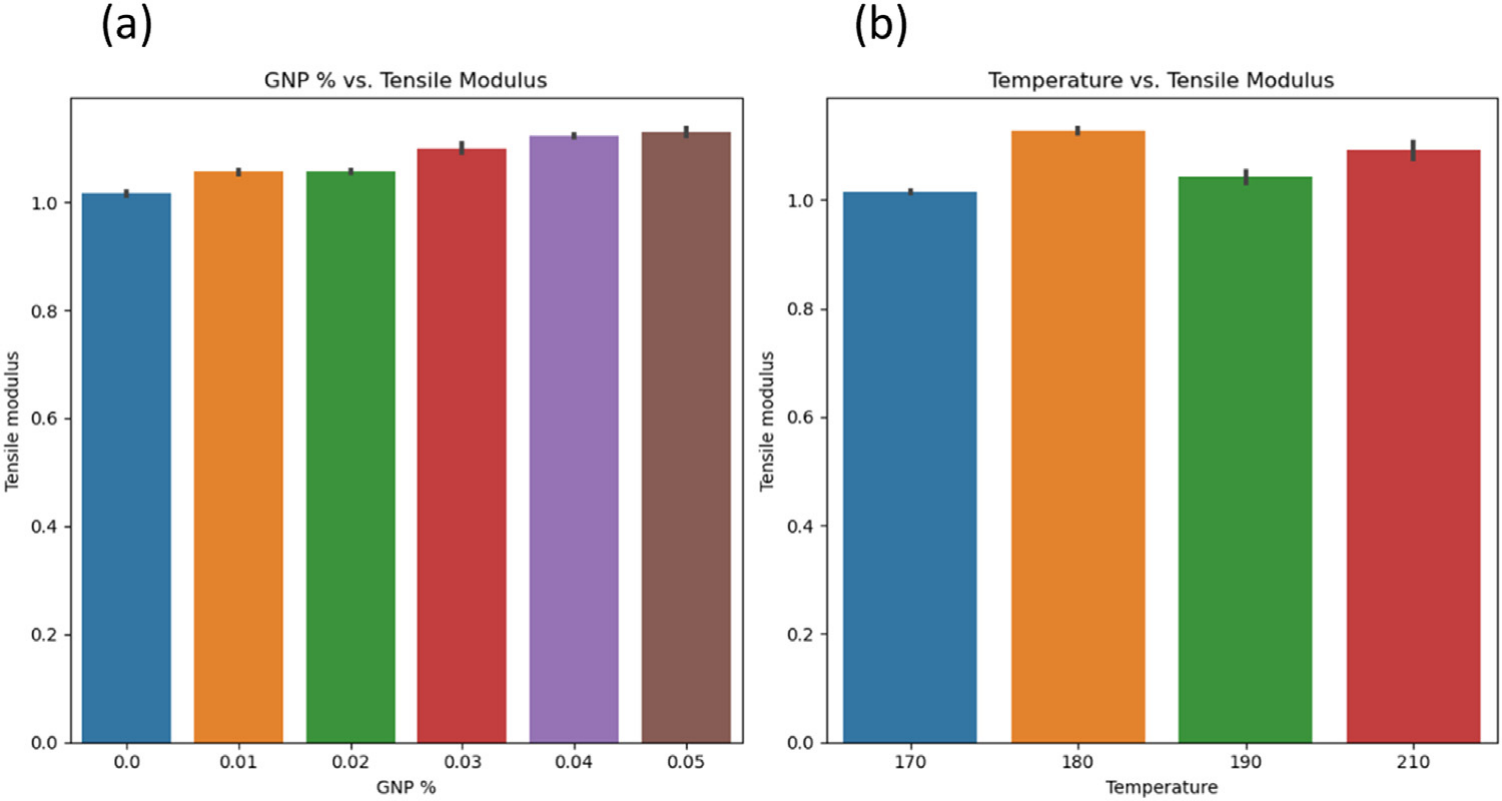
a) GNP vs. tensile modulus; b) temperature vs. tensile modulus.

**Fig. 6:**
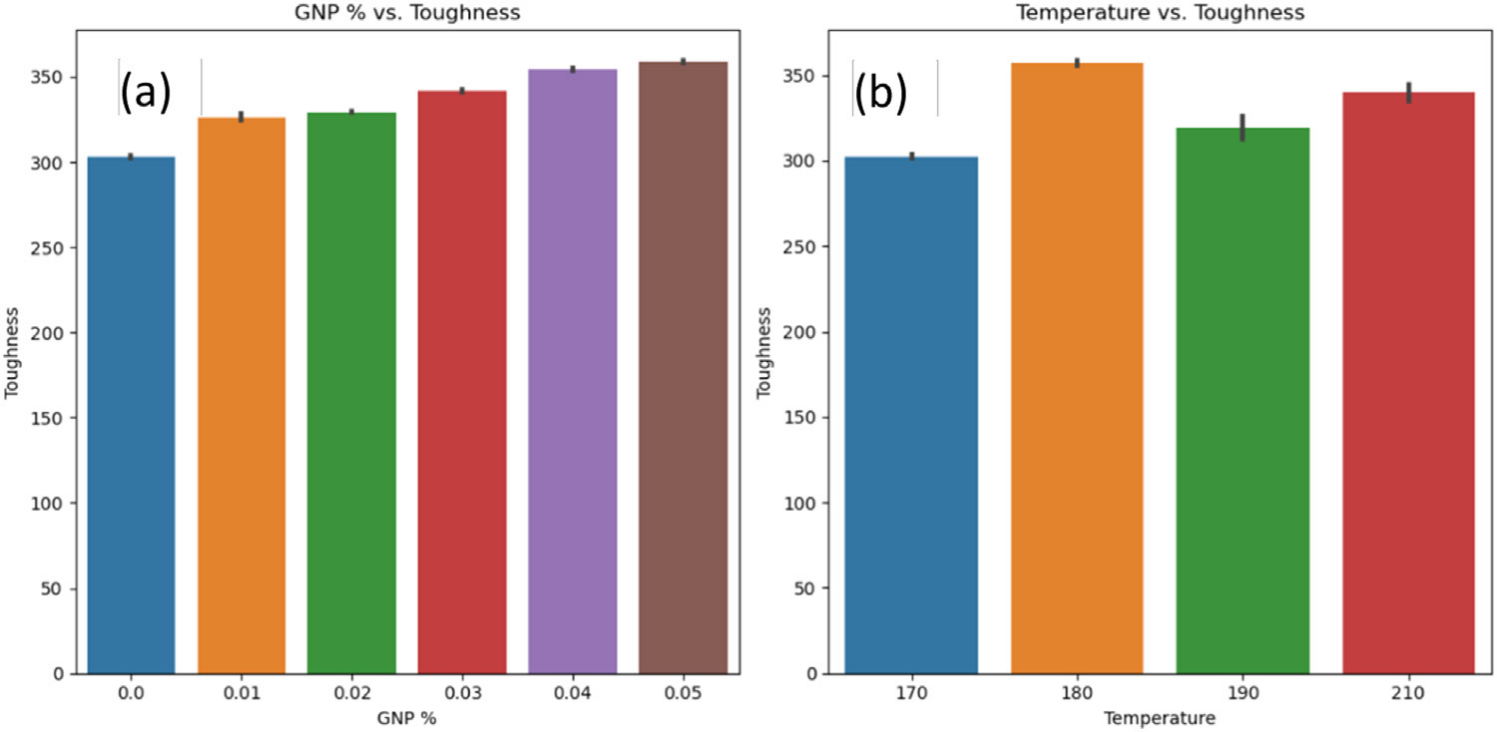
a) GNP vs. tensile modulus; b) temperature vs. tensile modulus.

**Fig. 7:**
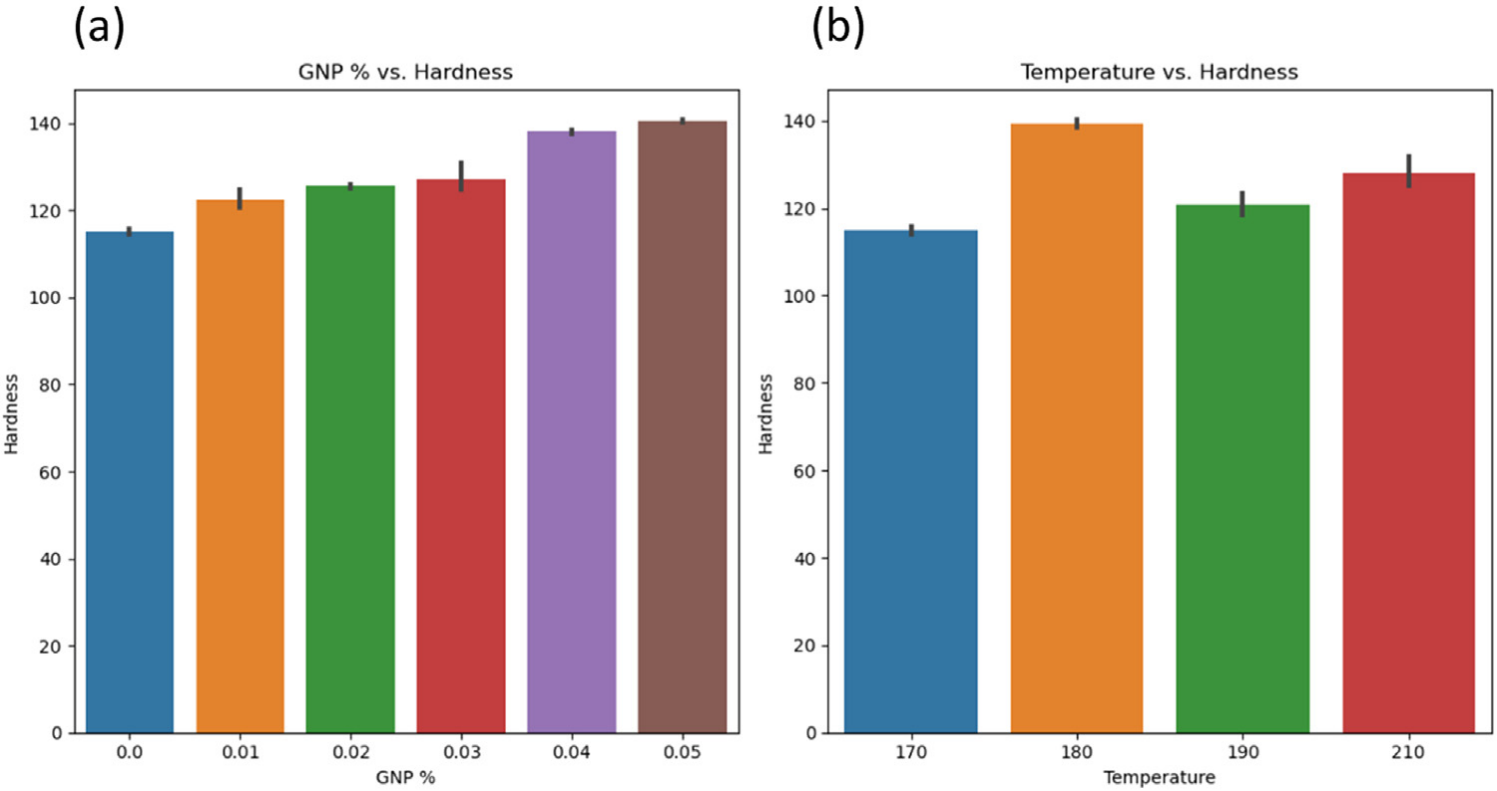
a) GNP vs. hardness; b) temperature vs. hardness.

**Fig. 8:**
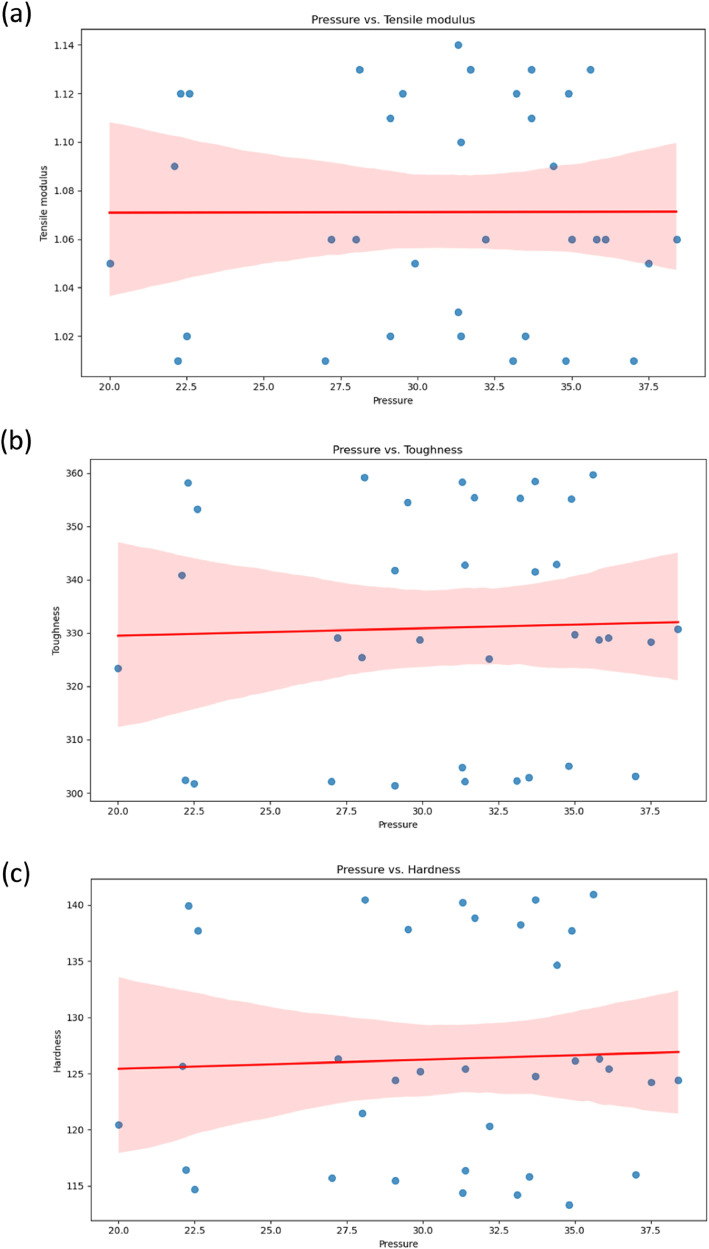
Pressure vs. a) tensile modulus; b) toughness; c) hardness.

Fig. 5a-b shows graphs between GNP % vs. Tensile Modulus and Temperature vs. Tensile modulus. GNP % significantly affects the tensile modulus [7,8], and it is difficult to derive the significance of temperature on the tensile modulus of HDPE. Fig. 6a-b shows a graph between GNP % vs. Toughness and Temperature vs. Toughness. GNP % significantly affects the toughness as well and a difficulty is noted while deriving effect of temperature on the tensile modulus of HDPE. Fig. 7a-b, shows the graph between GNP % vs. Hardness and Temperature vs. Hardness. An increase in GNP % increases the hardness of HDPE, while temperature doesn't have much significance. Similarly, Fig. 8a, b, and c shows the relationship between pressure and dependent variables (hardness, toughness, and tensile modulus). No strong correlation was observed with pressure as an independent variable. This was further substantiated by comparing the p values in OLS results, where these parameters were not significant.

OLS method does not yield a proper result due to the non-normality of data and autocorrelation. Hence, an advanced machine learning algorithm was used to carry out further predictive modeling.

### 4.5 Ensemble machine learning algorithm

A comparative analysis of different algorithms: Linear Regression, Ridge Regression, Lasso Regression, Elastic Net, Decision Tree, Random Forest, Gradient Boosting, Support Vector regression, and K-nearest neighbors was carried out [9]. The prediction was made for all the dependent variables: Tensile modulus, Toughness and, Hardness. The best model in terms of prediction accuracy was the Random Forest Regressor, followed by the Decision Tree regressor.

Random Forest regressor is an ensemble ML algorithm that can handle non-linearity, reduce overfitting, and is robust to noise and outliers with built-in cross-validation [10]. The initial model was built, and hyperparameter tuning was done using GridSearchCV and RandomSearchCV with the following parameters. The results are shown in Table 7, 8 and 9.

**Table 7:** Hyper parameters.

1. GridSearchCV	2. RandomSearchCV
**'n_estimators': [50, 100, 200],** **'max_features': ['auto', 'sqrt', 'log2′],** **'max_depth': [10, 20, 30, None],** **'min_samples_split': [2, 5, 10],** **'min_samples_leaf': [1, 2, 4]**	'n_estimators': np.arange(50, 300, 50), 'max_features': ['auto', 'sqrt', 'log2′], 'max_depth': [10, 20, 30, None], 'min_samples_split': np.arange(2, 11, 1), 'min_samples_leaf': np.arange(1, 5, 1)

**Table 8:** Cross validation GridSearchCV results.

	Result 1: Hyperparameters	Result 2: Cross Validation
**Tensile Modulus**	{'max_depth': None, 'min_samples_leaf': 1, 'min_samples_split': 2, 'n_estimators': 100}	[0.93009878 0.97290331 0.95378864 0.97906307 0.95239942] Mean R2 Score: 0.9576506437784189
**Hardness**	{'max_depth': None, 'min_samples_leaf': 2, 'min_samples_split': 5, 'n_estimators': 100}	[0.87423192 0.99540844 0.96189254 0.85702785 0.91477644] Mean R2 Score: 0.9206674381221867
**Toughness**	{'max_depth': None, 'min_samples_leaf': 1, 'min_samples_split': 2, 'n_estimators': 100}	[0.97407948 0.99431066 0.99192007 0.96552459 0.99075314] Mean R2 Score: 0.9833175874308614

**Table 9:** Cross validation RandomSearchCV.

	Result 1: Hyperparameters	Result 2: Cross Validation
**Tensile Modulus**	{'n_estimators': 100, 'min_samples_split': 5, 'min_samples_leaf': 1, 'max_depth': None}	[0.94292033 0.97317649 0.96229745 0.97861238 0.95181596] Mean R2 Score: 0.9617645221044034
**Hardness**	{'n_estimators': 100, 'min_samples_split': 5, 'min_samples_leaf': 2, 'max_depth': 20}	[0.87423192 0.99540844 0.96189254 0.85702785 0.91477644] Mean R2 Score: 0.9206674381221867
**Toughness**	{'n_estimators': 100, 'min_samples_split': 5, 'min_samples_leaf': 1, 'max_depth': None}	[0.98109576 0.98851818 0.99433055 0.97436278 0.98693249] Mean R2 Score: 0.985047949580317

**Fig. 9:**
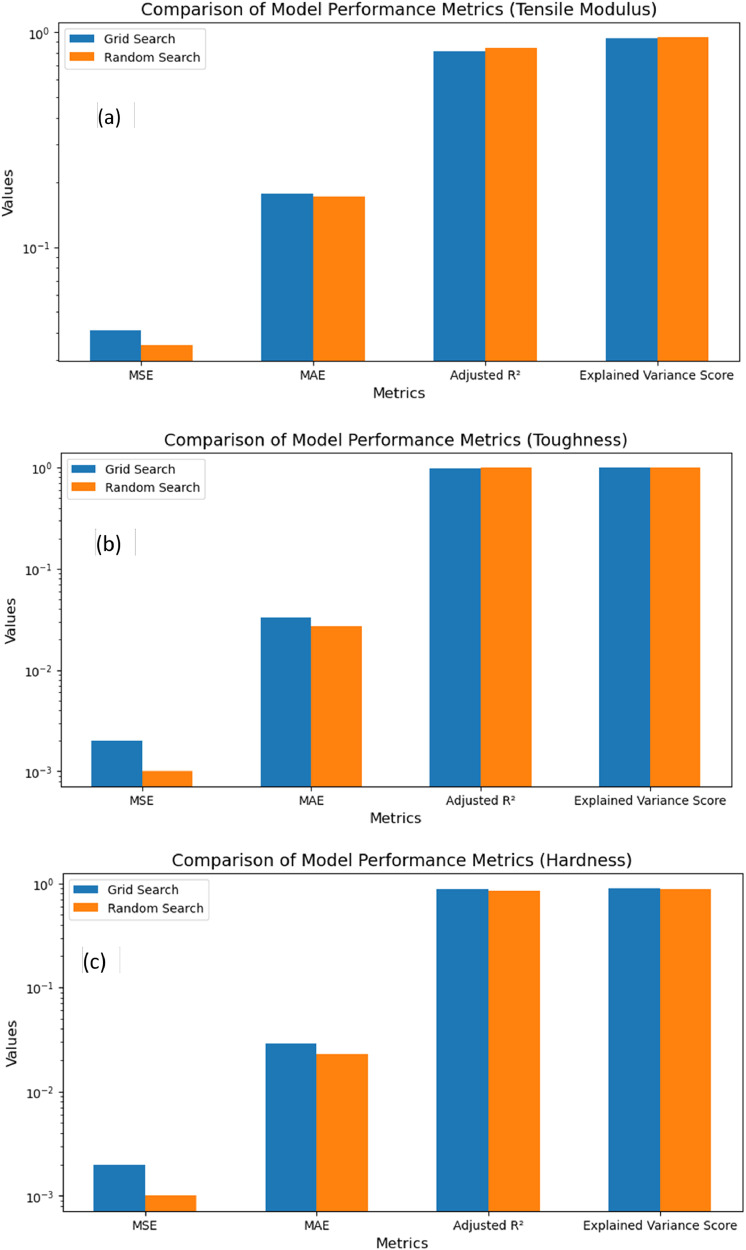
Performance evaluation matrix of a) tensile modulus; b) toughness; c) hardness.

Grid SearchCV and Random SearchCV both has appreciable results but Random search CV has proved to perform better for tensile modulus and toughness. For hardness, both are performing well. Other performance parameters like R^2^, Adj. R^2^, Mean Squared Error, Mean Absolute Error are shown in Fig. 9a, b, and c.

### 4.6 Feature of highest importance

**Fig. 10:**
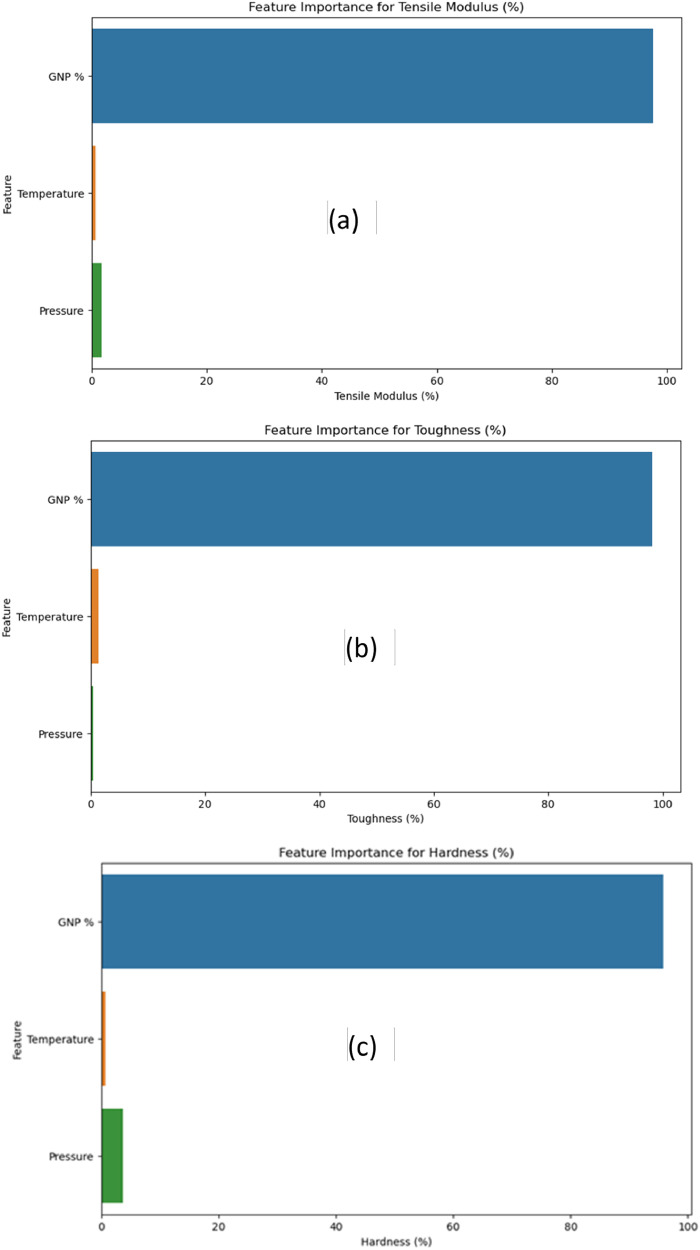
Feature importance for a) tensile modulus; b) toughness; c) hardness.

The feature of highest importance is calculated using the MDI-Mean Decrease in Impurity or Gini importance method as a part of the Random Forest Regressor. The following graph (as shown in Fig. 10a, b, c and Table 10) shows that GNP % has the highest and cardinal importance on dependent parameters.

**Table 10:** Feature of highest importance.

	Feature	Tensile Modulus (%)	Toughness (%)	Hardness (%)
**0**	GNP %	97.618925	98.208688	95.797200
**1**	Temperature	0.726963	1.391717	0.587676
**2**	Pressure	1.654112	0.399595	3.615125

### 4.7 Deriving rules for splitting

**Fig. 11:**
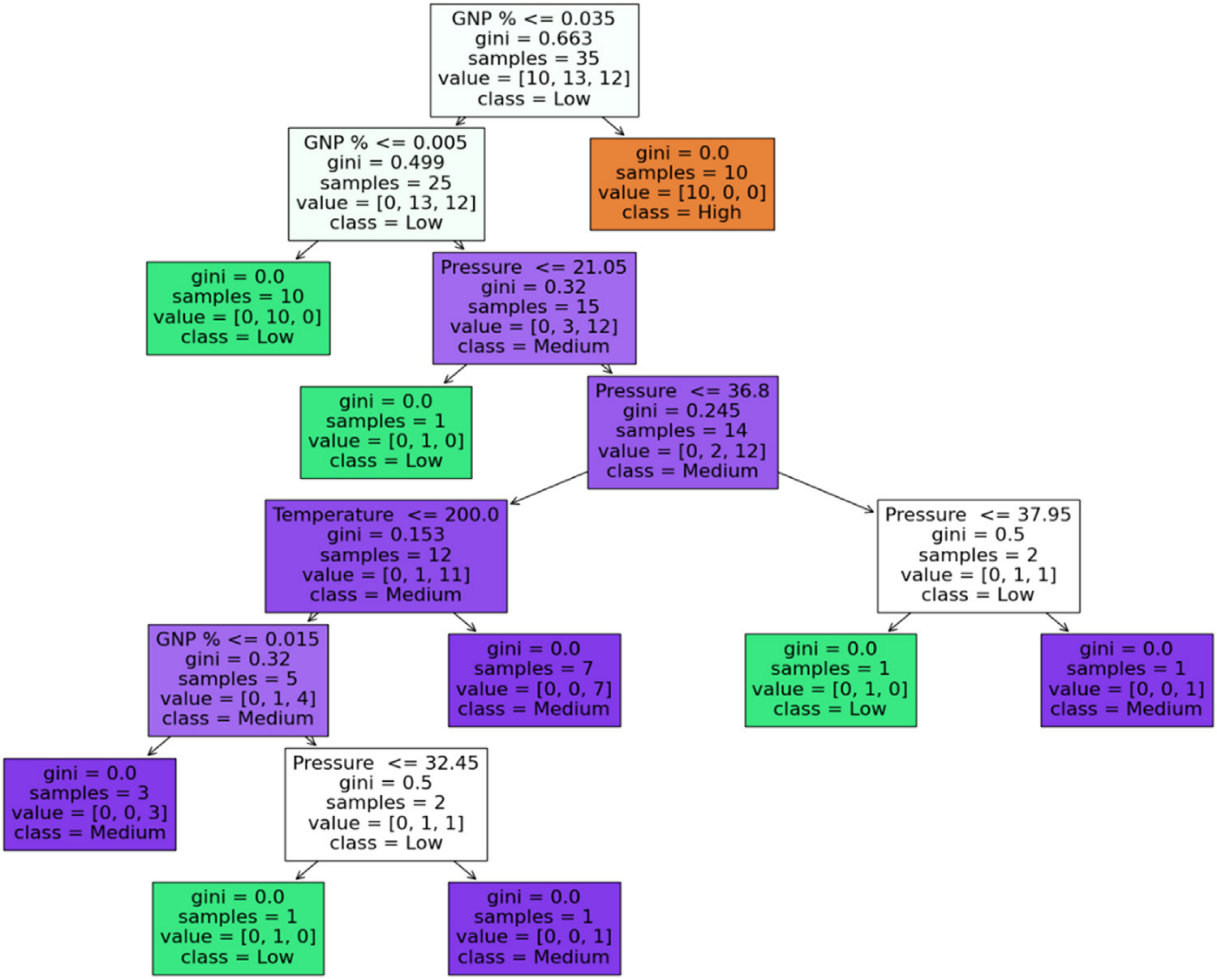
Decision tree for tensile modulus.

**Fig. 12:**
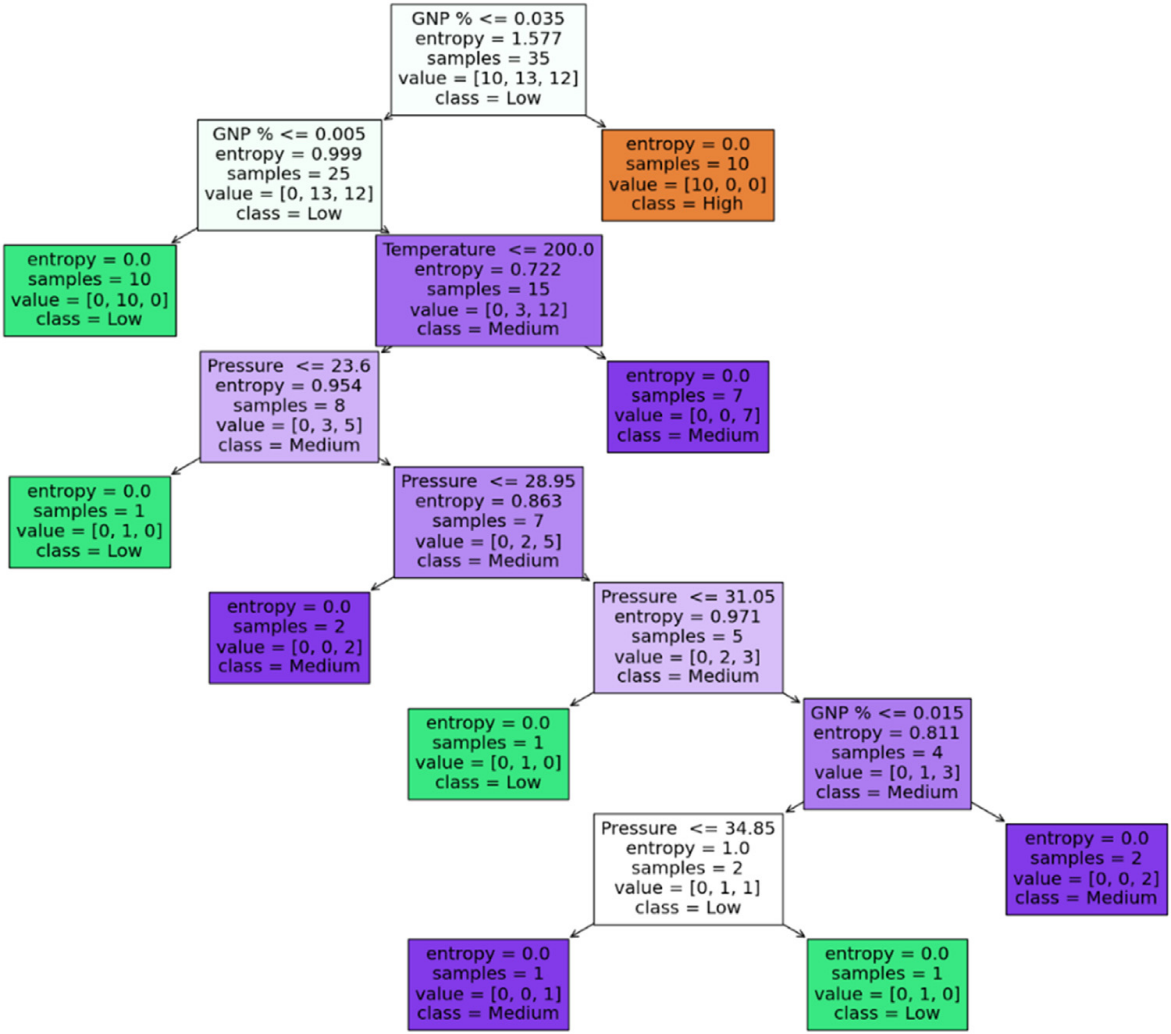
Decision tree for toughness.

**Fig. 13:**
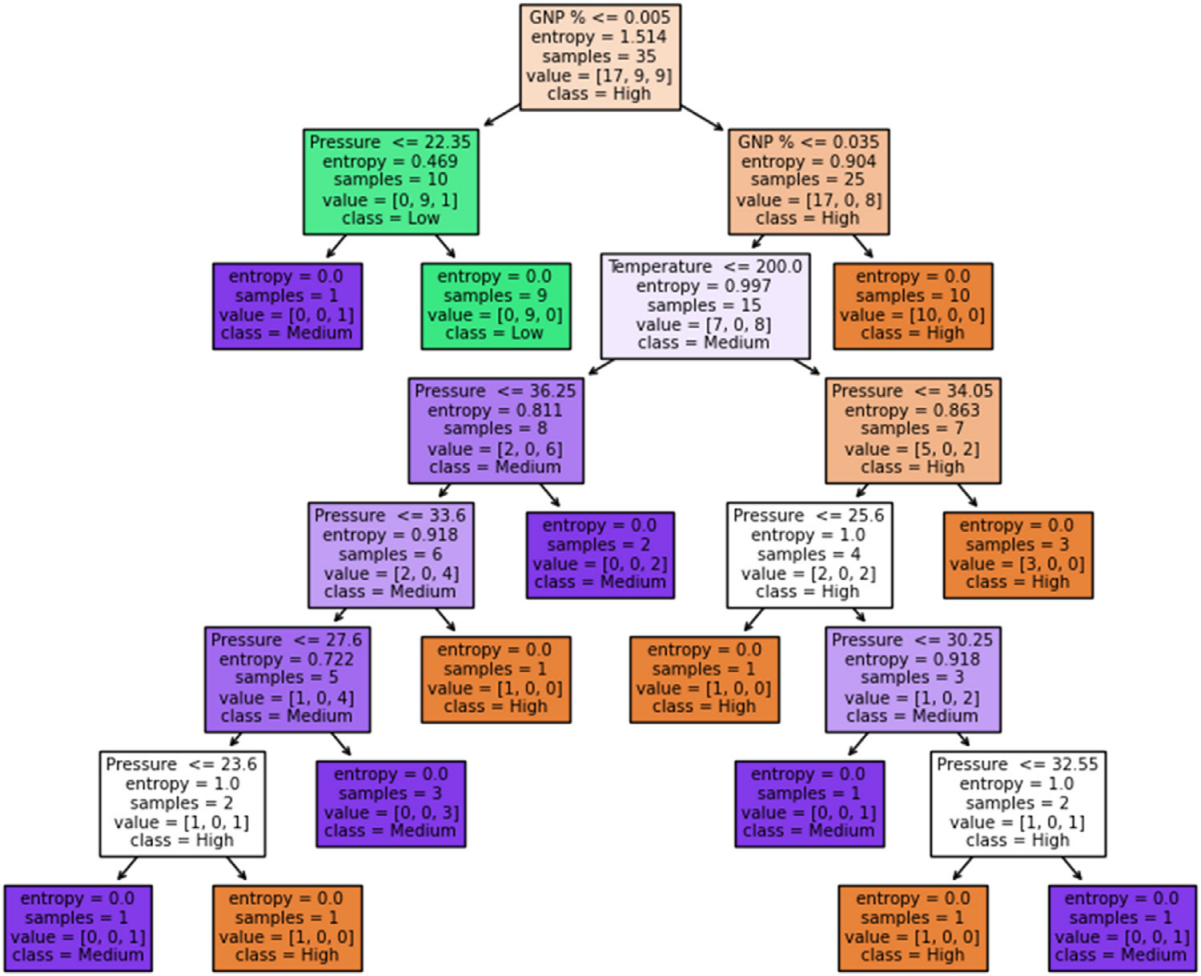
Decision tree for hardness.

The rules for splitting are derived using a decision tree classifier; the initial dataset for tensile modulus, toughness, and hardness was divided into three quartiles and labeled as "High," "Medium," and "Low." The rules help us understand which combination leads to high, medium, or low. Fig. 11, Fig. 12, and Fig. 13 delineate the decision tree, and Table 11, Table 12, and Table 13 expounds the splitting rules for each class.

**Table 11:** Tensile modulus (Gini).

Rule No.	Condition(s)	Predicted Class
**Rule 1:**	If GNP % ≤ 0.035	Low
**Rule 2:**	If GNP % ≤ 0.005	Low
**Rule 3:**	If GNP % > 0.005 and GNP % ≤ 0.035	Low
**Rule 4:**	If GNP % > 0.035	High
**Rule 5:**	If GNP % > 0.005 and GNP % ≤ 0.035 and Pressure ≤ 21.05	Medium
**Rule 6:**	If GNP % > 0.005 and GNP % ≤ 0.035 and Pressure > 21.05	Medium
**Rule 7:**	If GNP % > 0.005 and GNP % ≤ 0.035 and Pressure ≤ 21.05 and Temperature ≤ 200.0	Medium
**Rule 8:**	If GNP % > 0.005 and GNP % ≤ 0.035 and Pressure ≤ 21.05 and Temperature > 200.0	Low
**Rule 9:**	If GNP % > 0.005 and GNP % ≤ 0.035 and Pressure > 21.05 and Pressure ≤ 36.8	Medium
**Rule 10:**	If GNP % > 0.005 and GNP % ≤ 0.035 and Pressure > 21.05 and Pressure > 36.8	Low
**Rule 11:**	If GNP % > 0.005 and GNP % ≤ 0.035 and Pressure > 21.05 and Pressure ≤ 36.8 and GNP % ≤ 0.015	Medium
**Rule 12:**	If GNP % > 0.005 and GNP % ≤ 0.035 and Pressure > 21.05 and Pressure ≤ 36.8 and GNP % > 0.015	Low
**Rule 13:**	If GNP % > 0.005 and GNP % ≤ 0.035 and Pressure > 21.05 and Pressure > 36.8 and Pressure ≤ 37.95	Low
**Rule 14:**	If GNP % > 0.005 and GNP % ≤ 0.035 and Pressure > 21.05 and Pressure > 36.8 and Pressure > 37.95	Medium

**Table 12:** Toughness entropy.

Rule No.	Condition(s)	Predicted Class
**Rule 1:**	If GNP % ≤ 0.035	Low
**Rule 2:**	If GNP % ≤ 0.005	Low
**Rule 3:**	If GNP % > 0.005 and GNP % ≤ 0.035	Low
**Rule 4:**	If GNP % > 0.035	High
**Rule 5:**	If GNP % > 0.005 and GNP % ≤ 0.035 and Temperature ≤ 200.0	Medium
**Rule 6:**	If GNP % > 0.005 and GNP % ≤ 0.035 and Temperature > 200.0	Medium
**Rule 7:**	If GNP % > 0.005 and GNP % ≤ 0.035 and Temperature ≤ 200.0 and Pressure ≤ 23.6	Medium
**Rule 8:**	If GNP % > 0.005 and GNP % ≤ 0.035 and Temperature ≤ 200.0 and Pressure > 23.6	Medium
**Rule 9:**	If GNP % > 0.005 and GNP % ≤ 0.035 and Temperature ≤ 200.0 and Pressure ≤ 23.6 and Pressure ≤ 28.95	Medium
**Rule 10:**	If GNP % > 0.005 and GNP % ≤ 0.035 and Temperature ≤ 200.0 and Pressure ≤ 23.6 and Pressure > 28.95	Medium
**Rule 11:**	If GNP % > 0.005 and GNP % ≤ 0.035 and Temperature ≤ 200.0 and Pressure ≤ 23.6 and Pressure ≤ 28.95 and Pressure ≤ 31.05	Medium
**Rule 12:**	If GNP % > 0.005 and GNP % ≤ 0.035 and Temperature ≤ 200.0 and Pressure ≤ 23.6 and Pressure ≤ 28.95 and Pressure > 31.05	Low
**Rule 13:**	If GNP % > 0.005 and GNP % ≤ 0.035 and Temperature ≤ 200.0 and Pressure ≤ 23.6 and Pressure > 28.95 and GNP % ≤ 0.015	Medium
**Rule 14:**	If GNP % > 0.005 and GNP % ≤ 0.035 and Temperature ≤ 200.0 and Pressure ≤ 23.6 and Pressure > 28.95 and GNP % > 0.015	Medium

**Table 13:** Hardness-entropy.

Rule No.	Condition(s)	Predicted Class
**Rule 1:**	If GNP % ≤ 0.005	High
**Rule 2:**	If GNP % > 0.005 and GNP % ≤ 0.035	High
**Rule 3:**	If GNP % > 0.035	High
**Rule 4:**	If GNP % > 0.005 and GNP % ≤ 0.035 and Pressure ≤ 22.35	Low
**Rule 5:**	If GNP % > 0.005 and GNP % ≤ 0.035 and Pressure > 22.35	Medium
**Rule 6:**	If GNP % > 0.005 and GNP % ≤ 0.035 and Pressure > 22.35 and Pressure ≤ 36.25	Medium
**Rule 7:**	If GNP % > 0.005 and GNP % ≤ 0.035 and Pressure > 22.35 and Pressure > 36.25	High
**Rule 8:**	If GNP % > 0.005 and GNP % ≤ 0.035 and Pressure > 22.35 and Pressure ≤ 36.25 and Temperature ≤ 200.0	Medium
**Rule 9:**	If GNP % > 0.005 and GNP % ≤ 0.035 and Pressure > 22.35 and Pressure ≤ 36.25 and Temperature > 200.0	High
**Rule 10:**	If GNP % > 0.005 and GNP % ≤ 0.035 and Pressure > 22.35 and Pressure ≤ 36.25 and Pressure ≤ 33.6	Medium
**Rule 11:**	If GNP % > 0.005 and GNP % ≤ 0.035 and Pressure > 22.35 and Pressure ≤ 36.25 and Pressure > 33.6	Medium
**Rule 12:**	If GNP % > 0.005 and GNP % ≤ 0.035 and Pressure > 22.35 and Pressure ≤ 36.25 and Pressure ≤ 33.6 and Pressure ≤ 27.6	Medium
**Rule 13:**	If GNP % > 0.005 and GNP % ≤ 0.035 and Pressure > 22.35 and Pressure ≤ 36.25 and Pressure ≤ 33.6 and Pressure > 27.6	High
**Rule 14:**	If GNP % > 0.005 and GNP % ≤ 0.035 and Pressure > 22.35 and Pressure ≤ 36.25 and Pressure > 33.6 and Pressure ≤ 23.6	High
**Rule 15:**	If GNP % > 0.005 and GNP % ≤ 0.035 and Pressure > 22.35 and Pressure ≤ 36.25 and Pressure > 33.6 and Pressure > 23.6	Medium
**Rule 16:**	If GNP % > 0.005 and GNP % ≤ 0.035 and Pressure > 22.35 and Pressure > 36.25 and Pressure ≤ 34.05	High
**Rule 17:**	If GNP % > 0.005 and GNP % ≤ 0.035 and Pressure > 22.35 and Pressure > 36.25 and Pressure > 34.05	Medium

## Limitations

Not applicable.

## Ethics Statement

Authors affirm that they adhere to ethical guidelines for publishing. The present article does not include human subjects, animal experiments, or data obtained from social media platforms*.*

## CRediT authorship contribution statement

**Nitesh Dhar Badgayan:** Investigation, Data curation, Resources, Conceptualization, Methodology, Software, Formal analysis, Writing – original draft. **Santosh Kumar Sahu:** Conceptualization, Data curation, Methodology, Software, Formal analysis, Writing – original draft. **Avisek Kundu:** Investigation, Conceptualization, Methodology, Resources, Writing – original draft. **Seeboli Ghosh Kundu:** Investigation, Conceptualization, Methodology, Resources, Writing – original draft.

## Data Availability

Mendeley DataDataset on Graphite nanoplatelet enhanced HDPE composites: An ensemble machine learning approach tensile modulus, hardness and toughness (Original data)
